# A Dual-Colour Architecture for Pump-Probe Spectroscopy of Ultrafast Magnetization Dynamics in the Sub-10-femtosecond Range

**DOI:** 10.1038/srep22872

**Published:** 2016-03-15

**Authors:** C. S. Gonçalves, A. S. Silva, D. Navas, M. Miranda, F. Silva, H. Crespo, D. S. Schmool

**Affiliations:** 1 Departamento de Física e Astronomia and IFIMUP-IN, Faculdade de Ciências, Universidade do Porto, Rua do Campo Alegre 687, 4169-007 Porto, Portugal; 2 Department of Physics, Lund University, P.O. Box 118, SE-221 00 Lund, Sweden; 3 Laboratoire PROMES CNRS (UPR 8521), Université de Perpignan Via Domitia, Perpignan, France

## Abstract

Current time-resolution-limited dynamic measurements clearly show the need for improved techniques to access processes on the sub-10-femtosecond timescale. To access this regime, we have designed and constructed a state-of-the-art time-resolved magneto-optic Kerr effect apparatus, based on a new dual-color scheme, for the measurement of ultrafast demagnetization and precessional dynamics in magnetic materials. This system can operate well below the current temporal ranges reported in the literature, which typically lie in the region of around 50 fs and above. We have used a dual-colour scheme, based on ultra broadband hollow-core fibre and chirped mirror pulse compression techniques, to obtain unprecedented sub-8-fs pump and probe pulse durations at the sample plane. To demonstrate the capabilities of this system for ultrafast demagnetization and precessional dynamics studies, we have performed measurements in a ferrimagnetic GdFeCo thin film. Our study has shown that the magnetization shows a sudden drop within the first picosecond after the pump pulse, a fast recovery (remagnetization) within a few picoseconds, followed by a clear oscillation or precession during a slower magnetization recovery. Moreover, we have experimentally confirmed for the first time that a sub-10-fs pulse is able to efficiently excite a magnetic system such as GdFeCo.

Laser-induced femtosecond magnetism or femtomagnetism^1^ is a new research field in modern magnetism, whose origin is usually dated to 1996, when Beaurepaire *et al.*^2^ first showed that ultrafast 60 fs laser pulses could be used to demagnetize a Ni thin film and to study the evolution of the magnetization dynamics within the first few picoseconds after excitation. Since this important discovery, a wealth of research has been dedicated to the study of several crucial questions in this field, such as: how fast can the magnetization of a system be changed, how can the corresponding change be measured, how can it be controlled on an ultrafast time scale and what are the shortest excitation pulses that can affect ultrafast dynamical processes in magnetic systems.

Based on the premise that the photo-excitation of a magnetic system with ultrashort laser pulses significantly alters the thermodynamic equilibrium among the constituent degrees of freedom of the system (charge carriers, spins, and lattice), a variety of dynamical processes have been observed on an extremely fast timescale, which are inaccessible via thermal equilibrium transitions^1^,^2^,^3^,^4^,^5^,^6^,^7^,^8^. These provide essential insight into the understanding of fundamental transient magnetic phenomena, and have clear implications for future high-speed, multifunctional magneto-optical device technology^3^,^4^. Therefore, the experimental observation of magnetization dynamics, induced by pump-probe methods, has gained significant impetus with the use of femtosecond laser sources, permitting the manipulation of the magnetization of ferro- and ferrimagnetic materials^2^,^5^,^6^,^7^ and to study their related properties on timescales ranging from tens of femtoseconds to hundreds of picoseconds^8^,^9^,^10^,^11^.

However, the exact way in which laser pulses can change the collective spin ordering in the coherent regime, or induce a complete spin reversal or a magnetic phase transition on ultrafast times, is not completely understood. In this ultrashort temporal regime, the highly non-equilibrium processes and photo-excited coherences are the most interesting and least understood issues, since the relevant characteristic timescales are comparable to (or even shorter than) one single oscillation cycle of phonons and magnons, spin-dependent scattering times, and dephasing times^6^. This raises some fundamental questions with respect to the cornerstones of our current understanding of magnetism and phase transitions, including the microscopic origin of angular momentum conservation and the validity of the thermodynamic description of magnetism at these extremely short timescales^5^.

Although rapid advances in ultrafast optical methods have allowed the temporal resolution to be gradually improved, the most recent measurements have been routinely performed with a temporal resolution that is typically limited to few tens of fs (e.g., 50 fs in reference 5). This prevents the excitation of ultrafast processes at shorter timescales, and a lack of information is expected on timescales below that of the pulse width.

Moreover, the need to achieve shorter temporal resolutions has been recently asserted by Bossini *et al.*^12^ where it was demonstrated that a successful excitation of high-frequency magnons demands laser pulses shorter than 50 fs. They showed that the femtosecond spin dynamics triggered by the impulsive excitation of the two-magnon (2M) mode in an anriferromagnet such as KNiF_3_, is only accessible with using experimental pump-probe system with sub-20 femtosecond resolution.

In this paper we present a new system for nondegenerate pump-probe measurements of magneto-dynamic processes, hence minimizing potential contamination from the pump beam while achieving the highest temporal resolution for a system of this type. The hollow-core fibre (HCF) compressor behind our design is extremely broadband, from 450 to 1050 nm, with a Fourier-limit below 3 fs^13^. Via dichroic beamsplitting and careful dispersion compensation, we generate dual-colour pump and probe pulses with sub-10 fs durations. Our system has been fully optically characterized using autocorrelation and d-scan techniques^14^. To demonstrate the capability of our time-resolved magneto-optic Kerr effect (TR-MOKE) system, we measured the dynamical behaviour of a GdFeCo thin film, where we observed both ultrafast demagnetization (“fast”) and its related precessional dynamics (“slow”), in agreement with literature^3^,^4^,^6^,^15^,^16^,^17^. This work and results enabled us to attain the following two key objectives:

1. Demonstration of unprecedented sub-10-fs temporal resolution from a novel (dual-colour) TR-MOKE system, which opens the way to assessing information on dynamical processes taking place at timescales well below 50 fs.
2. Demonstration of sub-10-fs excitation: although the interaction of femtosecond laser pulses with spins, using Gaussian pulses of 10 fs width, has been theoretically studied by Vonesch *et al.*^18^, we have experimentally confirmed for the first time that an intense sub-10-fs pulse is able to efficiently excite a ferrimagnetic system such as a GdFeCo thin film.

## Experimental dual-colour pump-probe system

Previous to the present work, two main designs of pump-probe TR-MOKE systems have been employed: the first is based on a degenerate interaction, where both pump and probe beams have the same spectral bandwidth (or frequency range), even though they can have different polarizations; the second corresponds to a nondegenerate setup, where the pump and probe beams exhibit significantly different spectral bandwidths. One of the major disadvantages of a degenerate system lies in the fact that the strong pump beam can contaminate the signal obtained from the probe beam. This rise to a rapidly oscillating signal at the detector when the pump and probe pulses overlap in both space and time at the sample surface. This phenomenon is commonly referred to as a “coherent artifact”^19^,^20^.

In conventional dual-color systems, one or more optical parametric amplifiers (OPAs)^21^ are usually employed as a secondary source of spectrally tunable femtosecond pulses, with spectra typically ranging from the visible to the near-infrared. The temporal resolution of these techniques is determined by the available laser pulse width. OPAs are commercial systems with temporal regimes typically from the picosecond to tens of femtoseconds. In this latter case, OPAs are usually pumped by titanium:sapphire lasers with a central wavelength of 800 nm (or by their second-harmonic at 400 nm), with pulse energies ranging from 0.5 to 1 mJ and compressed pulse durations from 20 to 100 fs^22^. Here we present a new methodology for the development of a dual-colour pump-probe setup that eliminates contamination of the pump beam and provides laser pulses with spectral width larger than previous techniques and allowing the generation of sub-10-fs pump and probe pulses (described in detail in the following sections).

## Architecture of the novel dual-colour system

**Figure 1:**
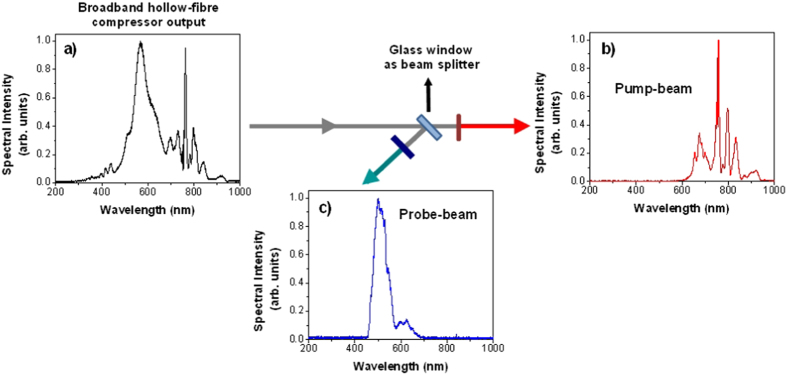
The spectral ranges of (**b**) the pump (transmitted) and (**c**) probe (reflected) beams after a fused silica window and colors filters, as well as (**a**) the full spectral range after the hollow fibre compressor and before beam splitting.

Our methodology consists in obtaining the two-colour pump and probe pulses from the same ultra-broadband hollow fibre source. Unlike OPA systems, this approach has several advantages, namely the fact that both pump and probe pulses have very broad bandwidths capable of supporting durations below 10 fs (Fig. 1).

In our experimental set-up, we have used a thin window that transmits 96% of the pulse energy to be used as the pump pulse, while the remaining 4% is reflected and used as the probe beam. The spectral overlap between the pump and probe spectra pulses is then reduced using optical bandpass filters. An advantage of this configuration is that by switching the two filters, the roles of the pulses can be exchanged when studying specific samples that may benefit from a particular combination of central wavelengths for the pump and the probe pulses. Moreover, the full spectrum of the broadband source can be used for both pump and probe pulses with only small changes in the setup (namely by removing the spectral filters), which is expected to increase the temporal resolution to below 5 fs. In this case, the setup will correspond to a degenerate system, albeit one with extremely high resolution.

## Experimental Apparatus

**Figure 2:**
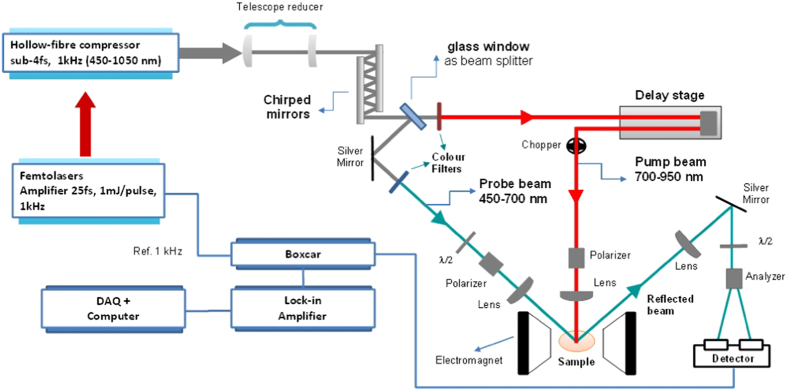
Schematic of the dual-colour pump-probe TR-MOKE apparatus.

Our pump-probe system (see Fig. 2) is based on a commercial titanium:sapphire laser amplifier (Femtolasers Compact Pro CE-phase) delivering sub-30-fs laser pulses (approximately 40 nm bandwidth centered at 800 nm) with 1 mJ of energy at a repetition rate of 1 kHz and with carrier-envelope phase (CEP) stabilization. These pulses are further post-compressed in a home-built state-of-the-art HCF and chirped-mirror compressor that uses the dispersion-scan (d-scan) technique for the simultaneous measurement of the pulses (as described in sections C and D)^13^,^14^,^23^. We make use of a refractive telescope, consisting of two lenses with focal lengths of 100 and 400 mm, to reduce the diameter of the beam prior to sending the pulses through a second chirped-mirror set (Fig. 2). These chirped mirrors effectively compensate the dispersion induced by the various optical elements along the beam paths. They have a working spectral bandwidth between 500 and 950 nm and are capable of compensating, up to high-order, the material dispersion associated with the telescope, the air paths and all remaining optical components (beamsplitter, filters, lenses, wave-plates and polarizers) traversed by the pulsed beams on their way to the sample under study.

After the chirped mirrors, a fused silica window is used as a broadband beamsplitter that reflects 4% of the energy to be used as the probe pulse (Fig. 1). The rest of the beam is transmitted and used as the pump pulse. To limit the spectral bandwidth of the pump, we use a lowpass filter with transmission ranging from 700 to 950 nm, and it is sent through a mechanical delay line (with a minimum step size of 0.3 fs). Furthermore, in the optical path of the pump beam, a polarizer cube has also been added. The spectral range of the probe beam is selected using a color bandpass filter that transmits in the range of 450–650 nm. In order to obtain a more homogeneous polarization of the probe beam, a combination of a polarizer cube and half-wave plate is also introduced allowing for the control of both probe beam polarization angle and intensity. Therefore, the setup permits the control of polarization and energy of both pulses, allowing us to determine the polarization rotation and ellipticity.

An optical chopper operating at 500 Hz and synchronized with the laser pulse train provides the reference for the detection electronics, which include a boxcar integrator and a lock-in amplifier coupled to an analog-to-digital converter. In both pump and probe beams, we place lenses with 200 and 100 mm focal length which produce focused spots with diameters of about 350 and 180 μm, respectively. In any optical pumping experiment, these spots must be accurately focused and overlapped on the sample surface, which has been performed with the help of a CCD camera. The presently installed magnet and associated bipolar power supply can generate a magnetic field of up to 1 T at the sample plane.

The detection of the magnetic state of the sample is performed via the measurement of the magneto-optic Kerr effect (MOKE) signal obtained from the probe beam after reflection from the sample surface. In the experiments discussed in section III, and although other MOKE configurations are possible upon small adjustments of the sample geometry, we have used the longitudinal Kerr effect, where the rotation angle of the polarization is directly proportional to the in-plane magnetization contribution. While the pump beam was set at normal incidence, the probe beam was set to 45° in order to maximize the magneto-optical response. The reflected beam is then directed through a broadband half-wave-plate and an analyzer to separate (and balance) the s and p polarized components prior to measuring their corresponding intensities using a balanced amplified photodetector. The difference in the signals allows us to evaluate the relative magnetization state of the sample as a function of the time delay introduced between pump and probe beams.

## Hollow core fibre and chirped-mirror compressor

The generation of ultrashort high-energy pulses with femtosecond durations is normally achieved via two main techniques: chirped-pulse amplification (CPA), usually based on titanium:sapphire technology, and optical parametric chirped-pulse amplification (OPCPA)^24^.

By using such techniques, it is possible to obtain pulses with durations of the order of 20–100 fs. Although few-cycle 10 fs pulses have been generated from a CPA system by using specialized optics to minimize the gain narrowing effects in the laser amplification process^25^, these techniques are not commonplace and the final durations are still far from the single-cycle regime. On the other hand, several few-cycle OPCPA systems have been demonstrated, also making use of Ti:Sapph technology^24^. These systems are mostly designed for intense laser-matter interaction and high-harmonic generation, having high repetition rates of the order of 1 MHz and a relatively low energy per pulse, usually in the microjoule range^26^. They also tend to be complex in their construction compared to CPA systems.

**Figure 3:**
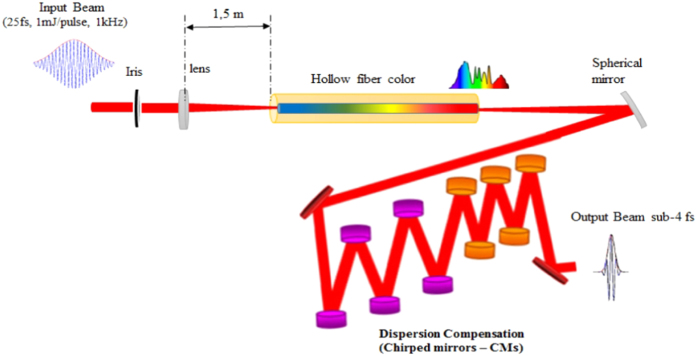
Simplified set-up of our hollow fibre and chirped mirror compressor. Although the chirped mirrors are identical, we used different colours to note that they should be placed at two different angles (5° and 19°).

In order to generate intense radiation, with pulse energies of hundreds of microjoules and durations in the sub-4-fs regime, as envisaged for versatile high resolution pump-probe experiments, a high-energy pulse compression technique based on pulses initially created by CPA, is employed. This technique is composed of two steps: i) nonlinear spectral broadening and ii) broadband dispersion compensation^13^,^27^,^28^ (Fig. 3). In the first step, the optical pulse spectrum is broadened inside a gas-filled hollow core fibre by self-phase modulation (SPM). The hollow-core fiber (HCF) compressor used in this setup comprises a standard 1 meter long hollow core fiber with a diameter of 250 micrometers and placed in a static pressure chamber with 1 atm of Argon. This is a quite standard configuration of HCF compressor in terms of fiber parameters and nominal gas pressure. The choice of Argon gas was motivated by its higher Kerr-nonlinearity compared to lighter noble gases such as Neon. We found that Argon gas was more effective for generating broadband, octave-spanning spectra with a well-behaved spectral phase when pumped with the sub-mJ and sub-30-fs pulses of our laser amplifier^13^. For higher energy and/or shorter pulse laser systems, other gases, such as Neon, can in principle be used too.

However, as the pulse spectrum broadens during nonlinear propagation along the hollow fibre, the pulse itself acquires a chirp due to SPM as well as due to dispersion in the gas and optical windows of the chamber containing the hollow fibre. This chirp needs to be compensated in order to obtain pulses with the shortest possible temporal duration. Dispersion compensation can be achieved in many ways; among the most common are prism pairs or chirped mirrors^29^. In our case, the 1 mJ, 27 fs pulses from our CPA amplifier are coupled into a hollow fibre through a lens with a focal length of 1.5 m (Fig. 3). The hollow-fibre is placed inside a chamber filled with Argon gas at a pressure of 1 bar. For input pulse energy of 500–700 μJ, the fibre transmission is approximately 40%. Dispersion compensation is performed ​​using a set of ultra-broadband chirped mirrors (CMs). The CMs (Ultrafast Innovations GmbH) are designed in such a way that when two reflections are combined, with incidence angles of 5° and 19°, respectively, the residual group delay oscillations are minimized^13^,^23^. This compressor consistently delivers sub-4-fs pulses, with 200–300 μJ energy and a stabilized carrier-envelope phase. Its output is extremely broadband, spanning from 450 to 1050 nm, and with a Fourier-limit below 3 fs^13^.

## Pulse characterization method: the d-scan technique

The time domain waveform and pulse width are key parameters in any study involving ultrashort optical pulses, since they determine the timescale of the excitation, and hence the interaction regime, as well as the achievable temporal resolution. Therefore, a precise knowledge of the optical pulse duration is fundamental. There are several techniques for ultrashort pulse characterization, from autocorrelation (AC)^30^, which can only provide an estimate of the pulse duration, to methods capable of retrieving the complete field (amplitude and phase) of the pulses, such as frequency-resolved optical gating (FROG)^31^ and spectral phase interferometry for direct electric-field reconstruction (SPIDER)^32^. In this work, our pulses were characterized by the new technique known as dispersion-scan, or d-scan^14^,^33^, which provides complete information about the ultrashort optical pulses^13^.

## The d-scan technique

The d-scan technique has been described in detail in references^13^,^14^,^33^, where it was used to successfully characterize the ultrashort laser pulses produced by the oscillator of our CPA system, as well as the output of the broadband hollow-core fibre compressor. In this work, d-scan was used to measure both the pump and probe pulses of our TR-MOKE system at the plane of the sample.

**Figure 4:**
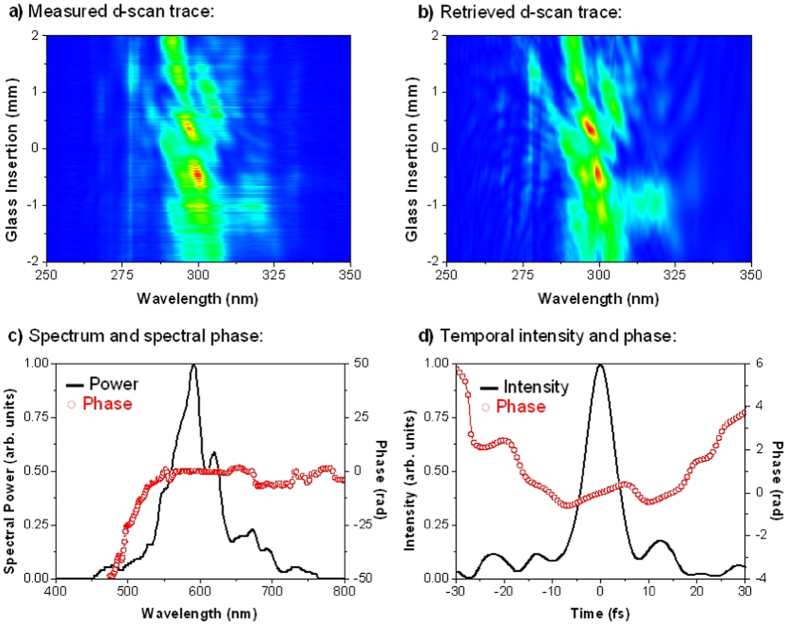
(**a**) Measured and (**b**) retrieved d-scan traces for the probe pulse (central wavelength of approx. 600 nm); retrieved probe pulse in the spectral (**c**) and temporal (**d**) domains; probe pulse width is 7.2 fs (FWHM).

The experimental implementation of d-scan consists in measuring the second-harmonic generation (SHG) spectrum of the pulse (or the spectrum of another nonlinear process) while the dispersion is varied via the insertion of one of two glass wedges around the point of maximum compression (minimum pulse duration). As a result, a spectrally resolved SHG trace as a function of wedge insertion (dispersion) is obtained (Fig. 4 a). Retrieval of the complete electric field of the pulses is then performed with an iterative numerical algorithm^14^ (Fig. 4 b). In the present set-up, we focus the pump (probe) pulses with a lens with focal length of 200 mm (100 mm) on a nonlinear crystal (Beta Barium Borate BBO, 20 μm thick, cut for type I SHG at 800 nm) placed in the position where the pump and probe pulses are overlapped on the sample. The SHG signal is collimated with a lens, and a blue filter is used to remove the remaining fundamental frequency signal before detection with an optical spectrometer (HR4000, Ocean Optics Inc.).

**Figure 5:**
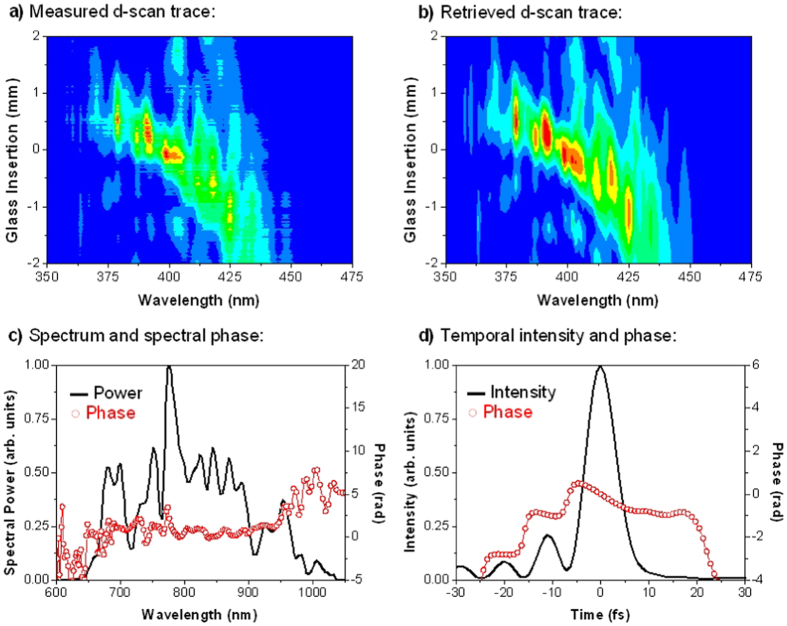
(**a**) Measured and (**b**) retrieved d-scan traces for the pump pulse (central wavelength of approx. 800 nm); retrieved pump pulse in the spectral (**c**) and temporal (**d**) domains; pump pulse width is 7.3 fs (FWHM).

Figures 4 and 5 show the measurements obtained for the probe and pump beams, with temporal durations of 7.2 and 7.3 fs, respectively. These demonstrate that dual-colour ultrashort pump and probe pulses with sub-10 fs duration have been successfully obtained at the plane of the sample, which ensures an ultra-high temporal resolution.

## Results and Discussion

In order to demonstrate the capabilities of our apparatus, we have studied the magnetic behavior of a ferrimagnetic rare earth–transition metal (RE-TM) thin film, in the form of a GdFeCo alloy. In this alloy, the Co and Fe sublattice magnetizations are coupled ferromagnetically with each other and the total magnetization of Fe and Co is coupled antiferromagnetically with that of Gd, yielding a ferrimagnetic order. This RE-TM alloy has been extensively studied due to its strong magneto-optical response^6^,^34^,^35^.

In particular, we carried out our measurements on a 20 nm thick Gd_23_Fe_68_Co_9_ thin film which was grown by magnetron sputtering in the following multilayer structure: glass/AlTi (10 nm)/SiN(5 nm)/GdFeCo(20 nm)/SiN(60 nm) (see References 16 and 17 for more details). The AlTi layer serves as a heat sink and the SiN as buffer and capping layers. The latter also serves as an antireflection coating. This film shows out-of-plane magnetic anisotropy and the Curie temperature (*T*_*C*_) is about ≈ 500 K^16^,^17^,^36^.

**Figure 6:**
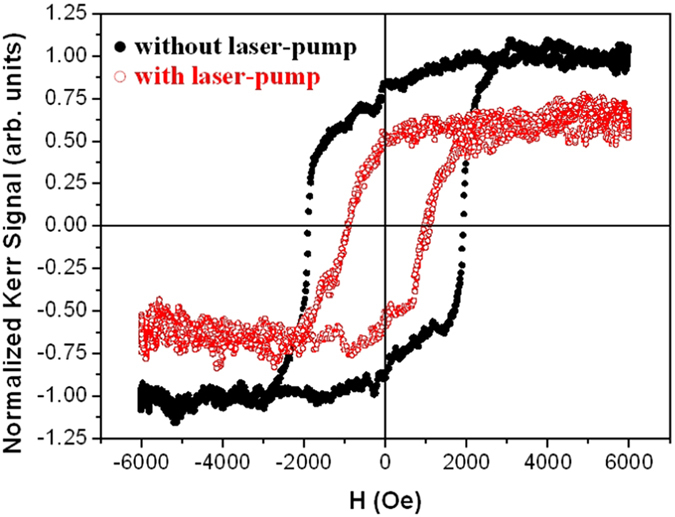
In-plane hysteresis loops of the GdFeCo thin film without (•) and with pump pulse (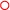) for a pump fluence of 2.7 mJ*/*cm^2^.

The laser-induced dynamics was firstly confirmed by measuring the in-plane hysteresis loops of the ferromagnetic GdFeCo thin film with and without applying the pump beam (Fig. 6). While in the latter case we obtain the standard static MOKE hysteresis loop, the curve, obtained in the presence of a pump fluence of 2.7 mJ*/*cm^2^ with a pulse width lower than 8 fs and for the time delay when the observed demagnetization was maximum, shows that the sample magnetization is reduced to about 70% of its normal saturation value.

Once we verified that the ferrimagnetic system could be excited using sub-10-fs pulses, we studied its time-resolved magnetization dynamics. However, we should note that with our set-up configuration, which uses linearly polarized laser pulses with a fluence of 2.7 mJ/cm^2^ and an in-plane external magnetic field, magnetization switching of the sample is not allowed and we can focus our attention on the ultrafast demagnetization and precessional processes^3^,^6^.

**Figure 7:**
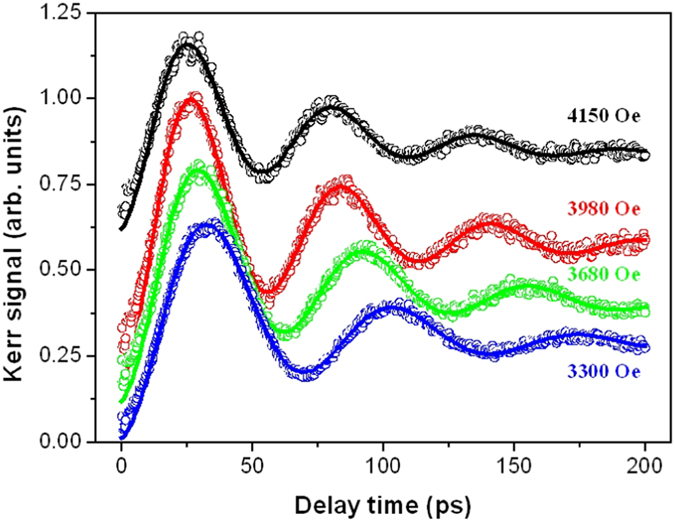
Time-resolved magneto-optical Kerr effect (TRMOKE) signals measured at a pump laser fluence of 2.7 mJ/cm^2^ for a 20-nm-thick GdFeCo film under different in-plane external applied fields. Theoretical curves (solid curves) are fit to the experimental data (open symbols) using Equation 1.

As was previously observed^16^,^37^, the magnetization shows a sudden drop within the first picosecond after the pump pulse, a fast recovery (remagnetization) within a few picoseconds, followed by a clear oscillation, or precession, during a slower magnetization recovery, which is characterized by the ferromagnetic resonance frequency *ω*_*FMR*_ and the Gilbert damping parameter *α*. Our TR-MOKE results are summarized in Fig. 7 where different in-plane external magnetic fields were applied to the sample.

We begin our analysis by focusing on the oscillatory behavior of the magnetization, as induced by the ultrafast-photo-excitation from the pump pulse. In our experiment, we applied an in-plane magnetic field strong enough to keep the sample magnetization in plane and parallel to the effective anisotropy field. The effective anisotropy field depends on several factors, such as the external applied magnetic field (strength and direction), the sample magnetocrystalline anisotropy energy and the shape anisotropy energy.

Initially, the pump beam energy is transferred to the electrons of the system and they are excited to above the Fermi level. Then, the electrons thermalize by electron-electron interactions, and transfer heat to the lattice by electron-phonon scattering and to the spin system by electron-spin scattering^1^,^2^. Therefore, the laser increases the spin temperature, modifying the strength of the magnetization, as well as the magnetocrystalline and shape anisotropy energies, and consequently inducing a change in the total effective anisotropy field. As this change is fast, the magnetization vector will not be able to follow the net internal field and will start to precess around its new equilibrium^4^. The magnetization recovery is described by a damped precessional motion around the effective field direction on a timescale of hundreds of picoseconds, until the original state is regained, and with a resonance frequency, ω_FMR_. Time-domain measurements on the excited precession give quantitative information on anisotropy, switching, and damping phenomena. In order to obtain a correct fit of the precessional processes, without any contamination from the ultrafast demagnetization, the TR-MOKE measurements were fitted, after a delay time of 10 ps, to a damped-harmonic function superposed with an exponential decay background^10^,^38^:


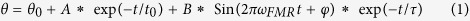


where *θ*_*0*_ and *A* are the background magnitudes and *t*_*0*_ is the background recovery time. The final term represents the precessional motion with the resonance frequency *ω*_*FMR*,_ relaxation time *τ* and initial phase *φ*. The solid curves in Fig. 7 are the fits using Equation 1, which show that the model agrees very well with the experimental data.

**Figure 8:**
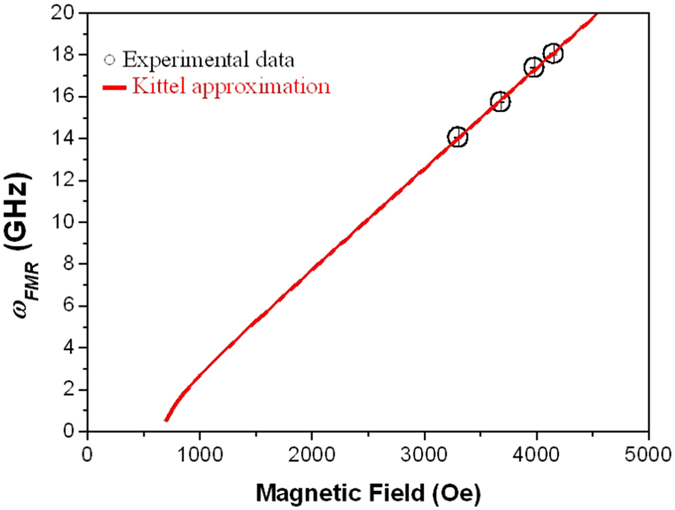
Field dependence of the resonance frequency (○) and the theoretical fit using the Kittel approximation (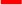).

Therefore, the resonance frequency of the precessional motion (*ω*_*FMR*_) can be determined^16^ and its field-dependence is shown in Fig. 8. Assuming that both the external magnetic field and the sample magnetization are in the sample plane, we obtained the field dependence of the resonance frequency, *ω*_*FMR*_*(H)*, from the Kittel approximation:


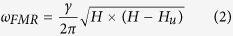


where *γ* is the gyromagnetic ratio and *H*_*u*_ is the effective anisotropy field of the sample. This analysis confirms that the GdFeCo thin film shows an effective out-of-plane anisotropy energy with an effective anisotropy field of *H*_*u*_ = (0.7 ± 0.2) kOe and an effective g-factor of *g*_*eff*_ = (3.4 ± 0.2), in agreement with the values obtained by Kato *et al.*^15^.

**Figure 9:**
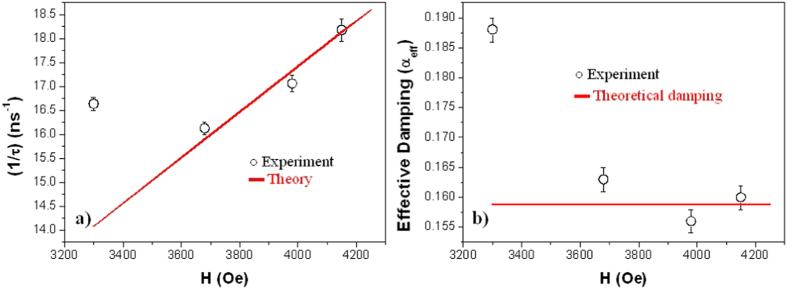
(**a**) Field dependence of (*1/τ*): Experimental data (○) and theoretical fit (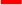) using equation (3). (**b**) Field dependence of (*α*_*eff*_): Experimental data (○) and theoretical damping coefficient (*α*) (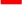) calculated from Equation (3).

Moreover, the variation of (*1/τ*) versus *H* were also obtained from the fits and compared with the theoretical calculations (Fig. 9 a)^10^:


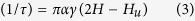


where *α* is the theoretical damping parameter, *H* the external applied field, *H*_*u*_ the effective anisotropy field and *γ* the gyromagnetic ratio. We obtained that the damping parameter is *α* = (0.159 ± 0.001), which is also in agreement with the values reported in other works^15^,^16^.

However, and although (*1/τ*) should be roughly proportional to *ω*_*FMR*_, a significant deviation from the theoretical curve has been observed at *H* = 3300 Oe. An alternative approach to evaluate *α* is based on defining the effective Gilbert damping parameter, *α*_*eff*_^10^:


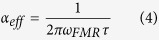


where *ω*_*FMR*_ and *τ* values at each applied *H* were estimated from the experiment (Fig. 9 b). The *α*_*eff*_ value is identical to or larger than *α*, and corresponds to an upper-bound value of *α*. As observed in Fig. 9 b, *α*_*eff*_ is constant for large applied magnetic fields, independent of H and close to *α* = (0.159 ± 0.001). However, at lower fields, *α*_*eff*_ is larger than *α*.

**Figure 10:**
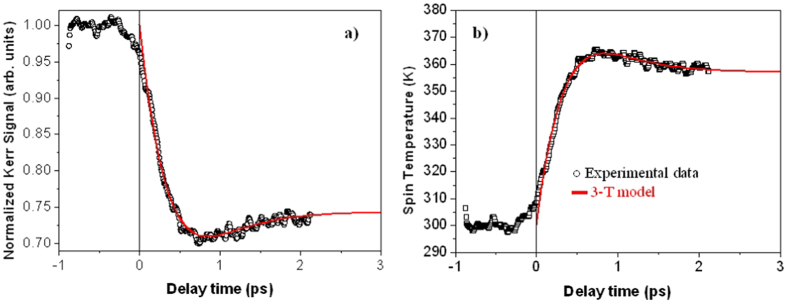
(**a**) Experimental demagnetization data of GdFeCo and (**b**) expected temporal evolution of the spin temperature using Equation 6 (open black symbols). Both curves were fitted within the 3T model (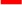).

We now focus our attention on the ultrafast demagnetization and the fast remagnetization processes within the first few picoseconds after excitation by the pump pulse. For this purpose, we have performed time-resolved pump-probe experiments using a pump pulse fluence of 2.7 mJ*/*cm^2^, with a pulse width of ~7.3 fs, an external applied field of 3300 Oe and a time delay from −1 to 2 ps. Under these experimental conditions, we previously determined that the sample was demagnetized by up to 70% of its normal saturation value (see Fig. 6). The normalized demagnetization signal is shown in Fig. 10 a. An ultrafast laser-induced demagnetization process is observed, with a minimum in the magnetization at a delay time of ≈800 fs, followed by its recovery, which is consistent with previous studies^17^,^39^.

Our analysis is based on the 3-Temperature model^1^,^2^ where it has been assumed that the energy supplied to the sample by the photons can be analyzed on different timescales via three different channels: the energy of the electrons (*e*), the phonon or lattice energy (*l*) and the spin energy (*s*), which is related with the magnetization change. Therefore, within the 3T model, the electron temperature, *T*_*e*_*(t)*, is coupled to the lattice temperature, *T*_*l*_*(t)*, and the spin temperature, *T*_*s*_*(t)*, in the form of the following three coupled differential equations^1^,^2^:


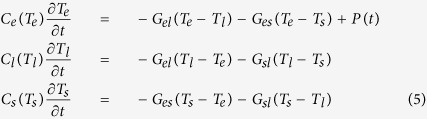


where *C*_*e*_*(T*_*e*_), *C*_*l*_*(T*_*l*_) and *C*_*s*_*(T*_*s*_) are the electronic, lattice and spin specific heats, respectively. *G*_*el*_, *G*_*es*_ and *G*_*sl*_ denote the electron-lattice, the electron-spin and the spin-lattice coupling constants, respectively, and determine the rate of the energy exchange between the electrons, the lattice and the spin baths. *P(t)* is the laser power density delivered to the electrons, which is described by a Gaussian function with a 8 fs pulse width (FWHM) and a fluence of 2.7 mJ*/*cm^2^.

In order to study the evolution of the magnetization, we first need to extract the experimental spin temperature (Fig. 10 b) which is achieved using the Bloch *T* ^3*/*2^ law^17^:


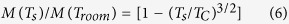


where *T*_*C*_ is the Curie Temperature (*T*_*C*_ = 500 K for GdCoFe)^17^,^36^. *T*_*s*_ reaches a maximum of around 365 K at t ≈ 800 fs after the pump pulse. Considering that the specific heat of the electrons is proportional to the electronic temperature^17^,^40^, *C*_*e*_*(T*_*e*_) = *cT*_*e*_ with *c* = 714 J/m^3^K^2^, and solving the three coupled differential equations (Eq. 5), the experimental spin temperature has been tuned until a reasonable agreement was obtained between the calculation and the experiment (see Fig. 10b). Our best fit was achieved for *C*_*l*_*(T*_*l*_) = (2.33 × 10^6^) J/m^3^K, *C*_*s*_*(T*_*s*_) = (0.34 × 10^4^) J/m^3^K, *G*_*el*_ = (8.19 × 10^17^) W/m^3^K, *G*_*es*_ = (0.2 × 10^16^) W/m^3^K and *G*_*sl*_ = (0.55 × 10^16^) W/m^3^K, which are reasonable values when compared with the typical values for metals^2^ and for a GdFeCo alloy with similar chemical composition^17^. Finally, and using Equation 6, the evolution of the magnetization is presented by the solid red line in Fig. 10 a.

In summary, we have developed a new dual-colour pump-probe MOKE spectrometer dedicated to the study of ultrafast demagnetization processes in a range of magnetic materials and structures. This versatile set-up uses state-of-the-art ultrafast optical methods to deliver ultrashort laser pump and probe pulses, at the sample position, permitting the observation of ultrafast magnetization dynamics at unprecedented temporal resolutions for a system of its type, well within the sub-10-fs range. This few-cycle regime is also highly promising for the direct excitation and observation of coherent ultrafast magnetodynamic behavior, which is expected to help us to gain a deeper understanding of both the conservation of angular momentum associated with the demagnetization process, and the relation between the spin thermalization and the corresponding quasi-equilibrium magnetization^5^. Furthermore, CEP-stabilized sub-5-fs pulses open the way for exploring the possible dependence of the induced magnetization dynamics on the electric field of the excitation pulses.

To demonstrate the essential capability of our dual-colour set-up, we have measured the ultrafast demagnetization and precessional dynamics in a ferrimagnetic GdFeCo thin film using a pump pulse fluence of 2.7 mJ*/*cm^2^, with sub-8-fs pulse width and ranging the time delay from −1 to hundreds of ps. We observed that the magnetization shows a sudden drop within the first picosecond after the pump pulse and a fast recovery (remagnetization) within a few picoseconds. This is followed by a clear oscillation or precession during a slower magnetization recovery. Regarding the precessional behaviour, we have determined both the ferromagnetic resonance frequency *ω*_*FMR*_ and the effective Gilbert damping parameter *α*_*eff*_, which are in good agreement with the current literature.

Furthermore, the ultrafast demagnetization and the fast remagnetization processes within the initial picoseconds after excitation by the pump pulse has been analyzed using an external applied field of 3300 Oe and a time delay ranging from −1 to 2 ps. Under these experimental conditions, measurements of the laser-induced demagnetization revealed a minimum at t ≈ 800 fs. Within the 3T model, the electronic, lattice and spin specific heats, as well as the electron-lattice, the electron-spin and the spin-lattice coupling constants were calculated.

Therefore, we have experimentally confirmed that our sub-8-fs pump pulses are able to efficiently induce demagnetization in a ferrimagnetic material and so we have established that the minimum pulse duration required for the demagnetization process is clearly in the sub-10-fs range. We expect that the present pump-probe system, when operating in a degenerate configuration with sub-5-fs CEP stabilized pulses, will enable us to further explore this limit, which we believe is an important aim for future developments in femtomagnetism.

## Additional Information

**How to cite this article**: Gonçalves, C. S. *et al.* A Dual-Colour Architecture for Pump-Probe Spectroscopy of Ultrafast Magnetization Dynamics in the Sub-10-femtosecond Range. *Sci. Rep.* **6**, 22872; doi: 10.1038/srep22872 (2016).

## References

[b1] Zhang G. P., Hübner W., Beaurepaire E. & Bigot J.-Y. In Topics of applied physics: spin dynamics in confined magnetic structures I (eds Hillebrands B. & Ounadjela K.). Laser-induced ultrafast demagnetization: Femtomagnetism, a new frontier?. Vol. 83, 245–289 (Springer-Verlag Berlin Heidelberg 2002).

[b2] Beaurepaire E., Merle J.-C., Daunois A. & Bigot J.-Y. Ultrafast spin dynamics in ferromagnetic nickel. Phys. Rev. Lett. 76, 4250–4253 (1996). 10061239 10.1103/PhysRevLett.76.4250

[b3] Stanciu C. D. *et al.* All-optical magnetic recording with circularly polarized light. Phys. Rev. Lett. 99, 047601 (2007). 17678404 10.1103/PhysRevLett.99.047601

[b4] Kirilyuk A., Kimel A. V. & Rasing T. Ultrafast optical manipulation of magnetic order. Rev. Mod. Phys. 82, 2731–2784 (2010).

[b5] Bigot J.-Y., Vomir M. & Beaurepaire E. Coherent ultrafast magnetism induced by femtosecond laser pulses. Nature Phys. 5, 515–520 (2009).

[b6] Kirilyuk A., Kimel A. V. & Rasing T. Laser-induced magnetization dynamics and reversal in ferrimagnetic alloys. Rep. Prog. Phys. 72, 026501 (2013). 23377279 10.1088/0034-4885/76/2/026501

[b7] Zhang G. P. & Hübner W. Laser-induced ultrafast demagnetization in ferromagnetic metals. Phys. Rev. Lett. 85, 3025–3028 (2000). 11005994 10.1103/PhysRevLett.85.3025

[b8] Hicken R. *et al.* Magnetooptical studies of magnetism on pico and femtosecond timescales. J. Magn. Magn. Mater. 242–245, 559–564 (2002).

[b9] Faahnle M., Seib J. & Illg C. Relating gilbert damping and ultrafast laser-induced demagnetization. Phys. Rev. B 82, 144405 (2010).

[b10] Mizukami S. *et al.* Fast magnetization precession observed in L10-FePt epitaxial thin film. Appl. Phys. Lett. 98, 052501 (2011).

[b11] Zhang Z. *et al.* Ultrafast laser-induced magnetization precession dynamics in FePt/CoFe exchange-coupled films. Appl. Phys. Lett. 97, 172508 (2010).

[b12] Bossini D. *et al.* Macrospin dynamics in antiferromagnets triggered by Sub-20 femtoseconds injection of nanomagnons. arXiv:1507.03377v1 [cond-mat.mes-hall]. 10.1038/ncomms10645 PMC4748265 26847766

[b13] Silva F. *et al.* Simultaneous compression, characterization and phase stabilization of GW-level 1.4 cycle VIS-NIR femtosecond pulses using a single dispersion-scan setup. Opt. Express 22, 10181–10191 (2014). 24921721 10.1364/OE.22.010181

[b14] Miranda M., Fordell T., Arnold C., L’Huillier A. & Crespo H. Simultaneous compression and characterization of ultrashort laser pulses using chirped mirrors and glass wedges. Opt. Express 20, 688–697 (2012). 22274393 10.1364/OE.20.000688

[b15] Kato T. *et al.* Compositional Dependence of *g*-Factor and Damping Constant of GdFeCo Amorphous Alloy Films. IEEE Trans. Magn. 44, 3380–3383 (2008).

[b16] Stanciu C. D. *et al.* Ultrafast spin dynamics across compensation points in ferrimagnetic GdFeCo: The role of angular momentum compensation. Phys. Rev. B 73, 220402(R) (2006).

[b17] Mekonnen A. *et al.* Role of the inter-sublattice exchange coupling in short-laser-pulse-induced demagnetization dynamics of GdCo and GdCoFe alloys. Phys. Rev. B 87, 180406(R) (2013).

[b18] Vonesch H. & Bigot J.-Y. Ultrafast spin-photon interaction investigated with coherent magneto-optics. Phys. Rev. B 85, 180407(R) (2012).

[b19] Luo C. W., Wang Y. T., Chen F. W., Shih H. C. & Kobayashi T. Eliminate coherence spike in reflection-type pump-probe measurements. Opt. Express 17, 11321–11327 (2009). 19582046 10.1364/oe.17.011321

[b20] Lebedev M. V., Misochko O. V., Dekorsy T. & Georgiev N. On the nature of coherent artifact. J. Exp. Theor. Phys. 100, 272–282 (2005).

[b21] Cerullo G. & De Silvestri S. Ultrafast optical parametric amplifiers. Rev. Sci. Instrum. 74, 1 (2003).

[b22] Steinmeyer G., Sutter D. H., Gallmann L., Matuschek N. & Keller U. Frontiers in ultrashort pulse generation: Pushing the limits in linear and nonlinear optics. Science 286, 1507–1512 (1999). 10567248 10.1126/science.286.5444.1507

[b23] Alonso B. *et al.* Characterization of sub-two-cycle pulses from a hollow-core fiber compressor in the spatiotemporal and spatio-spectral domains. Appl. Phys. B 112, 105–114 (2013).

[b24] Witte S. & Eikema K. S. E. Ultrafast optical parametric chirped-pulse amplification. IEEE J. Sel. Top. Quantum Electron. 18, 296–307 (2012).

[b25] Eilanlou A. A., Nabekawa Y., Ishikawa K. L., Takahashi H. & Midorikawa K. Direct amplification of terawatt sub-10-fs pulses in a CPA system of Ti:sapphire laser. Opt. Express 16, 13431–13438 (2008). 18711582 10.1364/oe.16.013431

[b26] Rothhardt J., Demmler S., Hädrich S., Limpert J. & Tünnermann A. Octave-spanning OPCPA system delivering CEP-stable few-cycle pulses and 22 W of average power at 1 MHz repetition rate. Opt. Express 20, 10870–10878 (2012). 22565712 10.1364/OE.20.010870

[b27] Nisoli M. *et al.* Compression of high-energy laser pulses below 5 fs. Opt. Lett. 22, 522–524 (1997). 18183254 10.1364/ol.22.000522

[b28] Schenkel B. *et al.* Generation of 3.8-fs pulses from adaptive compression of a cascaded hollow fiber supercontinuum. Opt. Lett. 28, 1987–1989 (2003). 14587798 10.1364/ol.28.001987

[b29] Szipöcs R., Ferencz K., Spielmann C. & Krausz F. Chirped multilayer coatings for broadband dispersion control in femtosecond lasers. Opt. Lett. 19, 201–203 (1994). 19829591 10.1364/ol.19.000201

[b30] Kärtner F. X. In MITopencourseware, Chapter 10: Pulse Characterization Ultrafast Optics, 333–370 (Spring Term 2005).

[b31] Trebino R. In Frequency-Resolved Optical Gating: The Measurement of Ultrashort Laser Pulses (Springer Science + Business Media, LLC, 2000).

[b32] C. Iaconis C. & Walmsley I. A. Spectral phase interferometry for direct electric-field reconstruction of ultrashort optical pulses. Opt. Lett. 23, 792–794 (1998). 18087344 10.1364/ol.23.000792

[b33] Miranda M. *et al.* Characterization of broadband few-cycle laser pulses with the d-scan technique. Opt. Express 20, 18732–18743 (2012). 23038514 10.1364/OE.20.018732

[b34] Mansuripur M. In The Physical Principles of Magneto-Optical Recording (Cambridge: Cambridge University Press, 1995).

[b35] Awano H. *et al.* Magnetic domain expansion readout for amplification of an ultra high density magneto-optical recording signal. Appl. Phys. Lett. 69, 4257 (1996).

[b36] Ostler T. A. *et al.* Crystallographically amorphous ferrimagnetic alloys: Comparing a localized atomistic spin model with experiments. Phys. Rev. B 84, 024407 (2011).

[b37] Hohlfeld J. *et al.* Fast magnetization reversal of GdFeCo induced by femtosecond laser pulses. Phys. Rev. B 65, 012413 (2001).

[b38] Song H.-S. *et al.* Observation of the intrinsic Gilbert damping constant in Co/Ni multilayers independent of the stack number with perpendicular anisotropy. Appl. Phys. Lett. 102, 102401 (2013).

[b39] Stanciu C. D. *et al.* Subpicosecond Magnetization Reversal across Ferrimagnetic Compensation Points. Phys. Rev. Lett. 99, 217204 (2007). 18233247 10.1103/PhysRevLett.99.217204

[b40] Kittel C. In Introduction to Solid State Physics (John Wiley & Sons, New York, 1996).

